# Risk factors for cardiac injury in patients with ischaemic stroke: a retrospective analysis

**DOI:** 10.3389/fneur.2025.1635972

**Published:** 2025-12-03

**Authors:** Hui Tang, Mengyuan Li, Zhi Liu, Daiquan Gao, Yunzhou Zhang, Biao Chen

**Affiliations:** 1 Department of Emergency, Xuanwu Hospital, Capital Medical University, Beijing, China; 2 Department of Radiotherapy, Peking Union Medical College Cancer Hospital, Chinese Academy of Medical Sciences, National Cancer Center, National Cancer Clinical Research Center, Beijing, China; 3 Department of Neurology, Xuanwu Hospital, Capital Medical University, Beijing, China

**Keywords:** ischaemic stroke, cardiac injury, reinfarction, Glasgow Coma Scale, blood urea nitrogen

## Abstract

**Objective:**

To identify independent risk factors for cardiac injury (CI) in patients with ischaemic stroke (IS) through a retrospective analysis, providing evidence for early screening and intervention strategies.

**Methods:**

A single-center retrospective study was conducted among hospitalized patients with IS, who were classified into CI and non-CI groups. CI was defined as elevation of one or more cardiac biomarkers (cTnI/T, CK-MB, or BNP) above the upper reference limit, with concurrent ECG or echocardiographic abnormalities. Clinical characteristics, laboratory parameters, and prognostic variables were analyzed using univariate methods (chi-square test, *t*-test, or Mann–Whitney *U* test), followed by multivariate logistic regression to identify independent risk factors. Odds ratios (ORs) and 95% confidence intervals were then calculated.

**Results:**

Across 393 patients with IS (100 with CI and 293 without CI), univariate analysis identified significant differences in multiple parameters, including age, vital signs, cardiac biomarkers (BNP, CK-MB, cardiac troponin I), inflammatory markers (hs-CRP, LDH), renal function (BUN, creatinine), coagulation markers (D-dimer), and comorbidities (atrial fibrillation, coronary heart disease, heart failure) between the groups (*p* < 0.05). However, after adjusting for these potential confounders in multivariate logistic regression analysis, neither Glasgow Coma Scale (GCS) scores nor blood urea nitrogen (BUN) levels remained statistically significant independent predictors of CI in patients with IS (*p* > 0.05).

**Conclusion:**

Although GCS scores and BUN levels may be associated with CI in patients with IS, a clear operational definition of CI is essential for diagnostic consistency and early risk identification. Enhanced screening and monitoring of high-risk patients, combined with clinical and biomarker evaluation, are essential for optimizing early management strategies and improving outcomes.

## 1 Introduction

Ischemic stroke (IS) is a leading cause of disability and mortality worldwide, primarily due to the acute disruption of cerebral blood flow, which causes damage to neural tissue (1). Recent evidence suggests that IS can also have systemic effects, particularly cardiac damage. Acute myocardial injury occurs in up to 25.3% of patients with IS (2, 3). The main manifestations of cardiac injury (CI) in these patients are myocardial damage, arrhythmias, and cardiac dysfunction (4), which may be related to autonomic dysfunction, inflammatory responses, thrombotic processes, and hypercoagulability (5, 6). In particular, dysregulation of the autonomic nervous system, especially sympathetic overactivation, can induce myocardial injury, arrhythmias, and haemodynamic instability (7).

Studies have shown that one third of the deaths following IS are due to heart damage. Nonfatal cardiac complications, including cardiac arrhythmias, myocardial ischemia, and left ventricular dysfunction, are also common following IS. In addition, post-stroke inflammation may further exacerbate myocardial injury, leading to poor patient prognosis or even death (6). Several biomarkers, including cardiac troponins I and T (cTnI/cTnT), brain natriuretic peptide (BNP), and high-sensitivity C-reactive protein (hs-CRP), have been used to assess the risk of CI in patients with IS (8). However, no consensus has been reached regarding the independent risk factors for CI in this population, and systematic analyses remain limited.

In clinical practice, early identification of patients with IS at high risk of CI is critical for optimizing treatment strategies and improving prognosis (9). Determining independent risk factors for CI would facilitate targeted cardiac monitoring strategies, improving diagnostic accuracy (10). Moreover, early intervention targeting modifiable risk factors, such as inflammation and coagulation abnormalities, could potentially mitigate cardiovascular complications (11). Tailored management approaches may increase survival rates and quality of life in stroke patients, ultimately optimizing overall therapeutic outcomes (12).

This study assesses the prevalence and current status of CI among patients with IS through retrospective analyses. It also identifies independent risk factors for CI in patients with IS using multivariate logistic regression models based on demographic characteristics, laboratory parameters, and other clinical variables to provide data-driven evidence to support early screening and intervention in the clinical setting.

## 2 Methods

This study was designed as a retrospective single-center cohort study to analyze the independent risk factors for CI in patients with IS. Clinical data were collected from the hospital information system and electronic medical records (EMR), and statistical analyses were conducted to evaluate the impact of several variables on CI in patients with IS.

### 2.1 Definition of cardiac injury (CI)

CI was operationally defined as biochemical evidence of myocardial injury accompanied by electrocardiographic (ECG) or echocardiographic (ECHO) abnormalities.

Biochemical criteria: elevation of one or more cardiac biomarkers above the institutional upper reference limit, including cardiac troponin I (cTnI > 0.04 ng/mL), cardiac troponin T (cTnT > 0.1 ng/mL), or B-type natriuretic peptide (BNP > 100 pg./mL).

The ECG abnormalities: new-onset arrhythmia, ST-segment deviation, T-wave inversion, or other ischaemic changes not present on baseline ECG.

The ECHO abnormalities: newly detected wall-motion abnormalities, structural changes (e.g., hypertrophy or thrombus), or left-ventricular systolic dysfunction (ejection fraction < 50%).

A diagnosis of CI required both biomarker elevation and at least one ECG or ECHO abnormality. Patients without biomarker elevation or cardiac abnormalities were classified as non-CI. This operational definition follows previously published criteria (2, 13).

### 2.2 Study population

Eligible patients were ≥18 years old with a confirmed IS diagnosis on neuroimaging (CT/MRI) and complete cardiac biomarker and ECG/ECHO data obtained upon admission. Patients were categorized into CI and non-CI groups according to the above definition.

Exclusion criteria included: acute myocardial infarction, chronic heart failure, severe arrhythmias, or other cardiac diseases; secondary CI due to infection, sepsis, or trauma; hospitalization < 48 h or incomplete data; and severe hepatic/renal dysfunction or malignancy.

### 2.3 Data collection

All clinical data were obtained from the EMR system. The collected variables included demographic characteristics (age, sex, body mass index [BMI], smoking history, and alcohol consumption); stroke-related factors (National Institutes of Health Stroke Scale [NIHSS] score, Glasgow Coma Scale [GCS] score, stroke location, Trial of Org 10,172 in Acute Stroke Treatment classification, and reinfarction within 3 months); laboratory parameters (blood urea nitrogen [BUN], creatinine, hs-CRP, lactate dehydrogenase [LDH], creatine kinase-MB, and D-dimer); and cardiac function indicators (ECG abnormalities, ECHO findings, and myocardial enzyme profiles). Medication use during hospitalization (e.g., antihypertensive agents, antiplatelet therapy, anticoagulants, statins, and hypoglycemic drugs) was reviewed when available. LDH and D-dimer were included based on established clinical evidence rather than exploratory analysis: LDH is a key marker of myocardial injury, with elevations reflecting cardiomyocyte necrosis and poor prognosis after ischemic stroke, while D-dimer is an indicator of coagulation and fibrinolysis activation, associated with large-vessel occlusion, cardiogenic embolism, and worse functional outcomes. Additionally, comprehensive information on patients’ comorbid conditions was collected, including hypertension, diabetes mellitus, coronary heart disease, atrial fibrillation, chronic obstructive pulmonary disease, rheumatic heart valve disease, history of intracerebral hemorrhage, old cerebral infarction, and transient ischemic attack, to evaluate their potential influence on cardiac injury.

### 2.4 Statistical analysis

Statistical analyses were performed using SPSS 26.0 software (IBM Corp., Armonk, NY, United States). Continuous variables following a normal distribution were expressed as mean ± standard deviation (Mean ± SD) and compared using the independent-sample *t*-test. Non-normally distributed data were presented as median (interquartile range) and analyzed using the Mann–Whitney *U* test. Categorical variables were analyzed using the chi-square test or Fisher’s exact test, as appropriate. Univariate logistic regression analysis was conducted to identify potential risk factors for cardiac injury (CI). To account for multiple comparisons and control the false discovery rate (FDR), *p*-values from univariate analyses were adjusted using the Benjamini-Hochberg procedure. Subsequently, variables with an FDR-adjusted *p*-value (*q*-value) < 0.1 in univariate analysis, together with clinically relevant covariates, were entered into a multivariate logistic regression model to determine independent predictors of CI in patients with ischemic stroke (IS). Results from both univariate and multivariate analyses were reported as odds ratios (ORs) and 95% confidence intervals (CIs). The variance inflation factor (VIF) was examined to assess multicollinearity, with a VIF < 10 indicating no severe multicollinearity. A two-tailed *p*-value <0.05 was considered statistically significant. The statistical power of the study, calculated using G*Power software based on the available sample size and effect size, ranged between 79 and 87%. Master of biostatistics (Liu SA) re-evaluated the statistical analysis used in this study and thought that they were reasonable. At the same time, the logistic regression model and Benjamini–Hochberg correction are reviewed to ensure the accuracy of the method and the robustness of the results.

### 2.5 Ethical statement

This study was approved by the institutional ethics committee. All patient data were anonymized to ensure privacy and data security. The study was conducted in accordance with the Declaration of Helsinki and relevant ethical guidelines.

## 3 Results

A total of 393 patients with IS were included in this study, including 100 patients with cardiac injury (CI) and 293 without CI. Univariate analysis of baseline characteristics revealed significant differences between the CI and non-CI groups in several parameters (Table 1). Patients in the CI group were significantly older and had higher NIHSS scores, heart rate, systolic blood pressure, as well as higher levels of BUN, creatinine, BNP, CK-MB, cardiac troponin I, hs-CRP, LDH, D-dimer, and glucose (all *p* < 0.05). They also had a higher prevalence of atrial fibrillation, coronary heart disease, and heart failure, and were more likely to have used oral anticoagulants and beta-blockers prior to admission.

**Table 1:** Baseline characteristics and univariate analysis of ischemic stroke (IS) patients with and without cardiac injury (CI).

Characteristic	Overall (*N* = 393)	Non-CI group (*n* = 293)	CI group (*n* = 100)	*P*-value
Demographics				
Age, years	67.0 ± 12.5	65.1 ± 12.8	72.8 ± 10.1	<0.001
Male sex, *n* (%)	237 (60.3)	180 (61.4)	57 (57.0)	0.443
BMI, kg/m^2^	25.1 ± 3.6	25.5 ± 3.5 (n = 42)	24.1 ± 3.8	0.187
Clinical scores				
NIHSS score	6 (3–11)	5 (2–9)	10 (5–15)	<0.001
GCS score	14 (12–15)	15 (13–15)	13 (10–14)	<0.001
Vital signs				
Systolic BP, mm Hg	152.5 ± 23.8	150.1 ± 22.5	159.8 ± 26.2	<0.001
Heart rate, beats/min	82.5 ± 15.7	80.8 ± 14.2	87.6 ± 18.5	<0.001
Comorbidities, *n* (%)				
Hypertension	286 (72.8)	208 (71.0)	78 (78.0)	0.173
Diabetes mellitus	149 (37.9)	107 (36.5)	42 (42.0)	0.332
Atrial fibrillation	48 (12.2)	30 (10.2)	18 (18.0)	0.041
Coronary heart disease	85 (21.6)	55 (18.8)	30 (30.0)	0.018
Heart failure	22 (5.6)	11 (3.8)	11 (11.0)	0.007
Hyperlipidemia	105 (26.7)	80 (27.3)	25 (25.0)	0.652
Old cerebral infarction	118 (30.0)	85 (29.0)	33 (33.0)	0.460
Chronic kidney disease	45 (11.5)	30 (10.2)	15 (15.0)	0.194
Medication use prior to admission, *n* (%)				
Antiplatelets (aspirin/clopidogrel)	125 (31.8)	98 (33.4)	27 (27.0)	0.232
Oral anticoagulants (warfarin/DOACs)	35 (8.9)	20 (6.8)	15 (15.0)	0.012
Beta-blockers	92 (23.4)	60 (20.5)	32 (32.0)	0.018
ACEI/ARB	158 (40.2)	120 (41.0)	38 (38.0)	0.602
Statins	110 (28.0)	75 (25.6)	35 (35.0)	0.067
Diuretics	48 (12.2)	32 (10.9)	16 (16.0)	0.183
Laboratory parameters				
Renal function				
Blood urea nitrogen, mmol/L	6.1 (4.8–7.9)	5.8 (4.6–7.2)	7.5 (5.6–9.8)	<0.001
Creatinine, μmol/L	76.9 ± 56.4	72.1 ± 41.2	90.8 ± 85.1	0.002
Cardiac biomarkers				
B-type natriuretic peptide, pg./mL	115 (48–308)	85 (36–178)	485 (210–1,250)	<0.001
Creatine kinase-MB, ng/mL	2.0 (1.3–3.1)	1.8 (1.2–2.5)	3.5 (2.2–8.0)	<0.001
Cardiac troponin I, ng/mL	0.01 (0.01–0.02)	0.01 (0.01–0.02)	0.08 (0.03–0.35)	<0.001
Inflammation and injury markers				
Hs-CRP, mg/L	5.2 (1.8–14.5)	3.8 (1.4–9.6)	12.7 (4.5–28.9)	<0.001
Lactate dehydrogenase, U/L	193 (162–240)	183 (156–218)	235 (195–305)	<0.001
Coagulation parameters				
D-dimer, mg/L	0.25 (0.16–0.58)	0.22 (0.15–0.45)	0.48 (0.22–1.35)	<0.001
Platelet count, ×10^9^/L	210.9 ± 69.7	215.3 ± 70.1	198.1 ± 67.5	0.031
Other				
Glucose, mmol/L	6.71 (5.66–8.90)	6.60 (5.60–8.30)	7.85 (6.20–11.20)	<0.001
Sodium, mmol/L	140.8 ± 3.5	141.0 ± 3.3	140.1 ± 3.9	0.027

Data presented as mean ± SD, median (IQR), or *n* (%). *P*-value from *t*-test, Mann–Whitney *U* test, or Chi-square test. BMI, Body Mass Index; NIHSS, National Institutes of Health Stroke Scale; GCS, Glasgow Coma Scale; BP, blood pressure; ACEI/ARB, Angiotensin-Converting Enzyme Inhibitors/Angiotensin II Receptor Blockers; DOACs, Direct Oral Anticoagulants; Hs-CRP, high-sensitivity C-reactive protein.

In contrast, no statistically significant differences were observed between groups for sex, BMI, diastolic blood pressure, history of hypertension, diabetes mellitus, hyperlipidemia, or the use of antiplatelets, ACEI/ARBs, statins, and diuretics (*p* > 0.05).

Following univariate analysis, variables with *q* < 0.1 (including GCS, BUN, atrial fibrillation, coronary heart disease, heart failure, and use of oral anticoagulants and beta-blockers) were entered into the multivariate logistic regression model. After adjusting for these potential confounders, neither GCS scores (OR = 0.94, 95% CI: 0.85–1.04, *p* = 0.221) nor BUN levels (OR = 1.02, 95% CI: 0.85–1.23, *p* = 0.812) in the 70–79 age group, or in any other age stratum, remained statistically significant independent predictors of CI (all adjusted *p* > 0.05, Table 2). The variance inflation factor for all variables in the models was <3, indicating no concerns regarding multicollinearity.

**Table 2:** Adjusted associations between predictors and cardiac injury, stratified by age groups.

Age group (years)	Variable	Crude OR (95% CI)	*P*-value	Adjusted OR (95% CI)	*P*-value
60–69	BUN (per 1 mmol/L)	1.25 (1.05–1.48)	0.011	1.08 (0.88–1.33)	0.465
	GCS (per 1 point)	0.82 (0.74–0.91)	<0.001	0.92 (0.82–1.03)	0.147
70–79	BUN (per 1 mmol/L)	1.18 (1.01–1.38)	0.038	1.02 (0.85–1.23)	0.812
	GCS (per 1 point)	0.88 (0.80–0.97)	0.008	0.94 (0.85–1.04)	0.221
≥80	BUN (per 1 mmol/L)	1.32 (1.02–1.71)	0.035	1.09 (0.80–1.48)	0.587
	GCS (per 1 point)	0.85 (0.74–0.98)	0.028	0.91 (0.78–1.06)	0.221

BUN, blood urea nitrogen; GCS, Glasgow Coma Scale. Adjusted Model: All analyses were adjusted for potential confounding variables, including history of atrial fibrillation, coronary heart disease, heart failure, and pre-admission use of oral anticoagulants and beta-blockers. The Crude OR represents the unadjusted effect from univariate analysis. The Adjusted OR represents the effect after controlling for the listed confounders in a multivariate logistic regression model.

## 4 Discussion

This study analyzed the risk factors for CI in patients with IS and found that GCS scores and BUN levels may be associated with the occurrence of CI, consistent with previous research findings (14, 15). Studies have shown that GCS scores serve as an indicator of neurological function (16), and elevated BUN levels may reflect renal dysfunction and systemic stress response (17), both of which could contribute to the development of CI in patients with IS (18).

### 4.1 Association between Glasgow Coma Scale score and cardiac injury

The GCS score is a critical indicator for assessing stroke severity, with lower scores typically reflecting more severe neurological impairment. Such impairment may lead to autonomic dysfunction and cardiovascular instability, thereby increasing the risk of CI (19). Previous studies have demonstrated a strong association between decreased GCS scores and an elevated risk of sudden cardiac death in patients with stroke (20). Although our findings did not demonstrate statistical significance, a non-significant trend was observed across age groups, suggesting a potential relationship between lower GCS scores and increased CI risk. These results support the clinical rationale for enhanced cardiovascular monitoring in patients with IS with marked neurological dysfunction (21). Healthcare professionals should dynamically assess GCS scores in patients with IS, be vigilant for signs of CI in patients with lower scores, and perform timely cardiac-related examinations, such as ECGs and cardiac enzyme profiles, to facilitate the early detection of CI.

### 4.2 Association between elevated blood urea nitrogen levels and cardiac injury

Blood urea nitrogen is a key marker for renal function assessment and an indicator of systemic stress (22). In patients with IS, elevated BUN levels may be associated with hypertension, dehydration, renal dysfunction, and systemic inflammatory responses, all of which can impact cardiac function and increase the risk of CI (23). Previous studies have also suggested a correlation between elevated BUN levels and an increased incidence of cardiovascular events. In our study, although elevated BUN was not a statistically significant predictor of CI in any age group, a consistent directional trend was observed. This suggests that monitoring renal function and fluid balance may be important in patients with IS, particularly those at risk of cardiovascular complications (14). Moreover, these patients should avoid the use of medications that may increase renal burden or affect myocardial contraction (e.g., non-steroidal anti-inflammatory drugs and certain antibiotics) to help prevent further BUN elevation and reduce the risk of cardiac events.

In addition, it should be noted that we excluded patients with missing critical clinical data and those with a hospital stay of less than 48 h. This approach was adopted to ensure data completeness and to allow sufficient time for the detection of myocardial injury markers. However, we acknowledge that such exclusions may introduce selection bias and reduce the representativeness of the sample. This potential bias has been addressed in the Limitations section, and we have proposed directions for improving future studies accordingly.

## 5 Limitations

Our findings are consistent with previous research suggesting a potential role for GCS scores and BUN levels in predicting post-stroke CI. However, unlike some studies, our multivariate analysis did not confirm these factors as independent predictors of CI, possibly due to sample size limitations or residual confounding variables. Differences in inclusion criteria, study design, and statistical methods across studies may also have contributed to the variations in the results. Future studies should include more sensitive cardiac biomarkers (e.g., high-sensitivity troponin and N-terminal pro-BNP) and include patients with IS with varying lengths of hospitalization to more accurately assess the factors influencing CI.

This study was conducted at a single center and used a retrospective approach, which may have introduced selection bias, limiting the generalisability of the findings to broader populations. Specifically, patients with missing critical clinical data and those hospitalized for less than 48 h were excluded to improve the accuracy and reliability of our results; however, these exclusion criteria may have reduced the representativeness of the study population and introduced selection bias. We have acknowledged this limitation and highlighted the need for future studies to include broader patient cohorts and evaluate its potential impact. The relatively small sample size may have resulted in insufficient statistical power, preventing some variables from demonstrating independent significance in multivariate analysis.

This study suggests that lower GCS scores and elevated BUN levels may be associated with CI in patients with IS, emphasizing the need for enhanced cardiac monitoring in high-risk patients. Combining these markers with other cardiac biomarkers may help refine early intervention strategies. Large-scale, multicentre studies to further validate the predictive value of GCS scores and BUN levels in IS-related CI and prospective cohort studies to eliminate retrospective study bias and strengthen causal inferences could be conducted. This could include the incorporation of additional biomarkers, including high-sensitivity CI markers, to enhance predictive accuracy and clinical applicability.

By addressing these aspects, future studies can provide stronger evidence for clinical decision-making, ultimately improving early detection and management of CI in patients with IS.

## 6 Conclusion

This study suggests that in patients with IS, the risk of CI may be influenced by clinical parameters such as GCS scores and BUN levels. Although neither variable reached statistical significance in stratified multivariate analysis across different age groups, the observed trends indicate the potential clinical relevance of these markers. Early dynamic assessment of accessible indicators such as GCS scores and BUN levels may facilitate the application of appropriate therapeutic measures (e.g., continuous ECG monitoring or restriction of fluid intake) to improve long-term survival and quality of life in patients with IS. Further large-scale, multicentre prospective studies are warranted to validate these results and identify reliable early predictors of CI in patients with IS.

## Data Availability

The original contributions presented in the study are included in the article/supplementary material, further inquiries can be directed to the corresponding authors.
